# Autism as a developmental disorder in intentional movement and affective engagement

**DOI:** 10.3389/fnint.2013.00049

**Published:** 2013-07-17

**Authors:** Colwyn Trevarthen, Jonathan T. Delafield-Butt

**Affiliations:** ^1^Department of Psychology, College of Humanities and Social Sciences, University of EdinburghEdinburgh, UK; ^2^Early Years, School of Education, Faculty of Humanities and Social Sciences, University of StrathclydeGlasgow, UK

**Keywords:** autism, motor development, emotional expression, communication, education, therapy

## Abstract

We review evidence that autistic spectrum disorders have their origin in early prenatal failure of development in systems that program timing, serial coordination and prospective control of movements, and that regulate affective evaluations of experiences. There are effects in early infancy, before medical diagnosis, especially in motor sequencing, selective or exploratory attention, affective expression and intersubjective engagement with parents. These are followed by retardation of cognitive development and language learning in the second or third year, which lead to a diagnosis of ASD. The early signs relate to abnormalities that have been found in brain stem systems and cerebellum in the embryo or early fetal stage, before the cerebral neocortex is functional, and they have clear consequences in infancy when neocortical systems are intensively elaborated. We propose, with evidence of the disturbances of posture, locomotion and prospective motor control in children with autism, as well as of their facial expression of interest and affect, and attention to other persons' expressions, that examination of the psychobiology of motor affective disorders, rather than later developing cognitive or linguistic ones, may facilitate early diagnosis. Research in this area may also explain how intense interaction, imitation or “expressive art” therapies, which respond intimately with motor activities, are effective at later stages. Exceptional talents of some autistic people may be acquired compensations for basic problems with expectant self-regulations of movement, attention and emotion.

## Introduction to a different, psychobiological approach

**“Generality of the problem of Syntax**: Not only speech, but all skilled acts seem to involve the same problems of serial ordering … Analysis of the nervous mechanisms underlying order in the more primitive acts, may contribute ultimately to the resolution even of the physiology of logic.”

(Lashley, 1951, pp. 121–122)

**“A Different Approach to the Problem**: In so far as an organism perceives a given object, it is prepared to respond with reference to it. This preparation-to-respond is absent in an organism that has failed to perceive.”

(Sperry, 1952, p. 296)

Lashley (1951) and Sperry (1952) observed that perception, intelligent action and thinking depend upon impulses that move the body purposefully. The animal brain contributes systematic and serial organization, in time and space, to muscle activity under expectant perceptual and emotional control. It is always active, not passively reactive to stimuli. Nor is the human brain ever animated by thoughts of external events alone. All mental and behavioral skills depend on preparation to respond with serial ordering of acts. “The sole product of brain function is motor coordination” (Sperry, 1952, p. 297). This is a psychobiological theory of motives and affects in the mind, clearly articulated before the advent of the “cognitive revolution” that divorced mind from vital body in the 1960's (Miller, 2003).

The motor theory of consciousness was inspired by the research of Charles Sherrington (1906) on “the integrative action of the nervous system.” It has support from developmental neurobiology and neuroembryology (Trevarthen, 1986a; Prechtl, 2001), from ethology of the adaptive action patterns of animals and how they communicate emotional evaluations for social cooperation (Gallistel, 1980; Marler, 1984; Fentress and Gadbois, 2001; Panksepp, 2005), and from infant psychology and communication (Trevarthen, 1986b, 2001a, 2009a; Stern, 2000, 2010).

Research focused on cognitive disorders of perceptual information processing, selective awareness, and representational thinking articulated in language, all of which skills develop after infancy—disregards the developmental foundations of experience in motor coordination, and in the expression of vital states as emotions for regulation of social life. In an animal's perceived world, its “Umwelt” (von Uexküll, 1957), conceptions of objects are created by the intentional subject's attempts to locate and perceive “sign stimuli” detected in the environment by dedicated receptors (Buchanan, 2008; Berthoz and Christen, 2009). Self-regulation of knowing, with emotional assessments of risks and benefits, becomes in humans the source of cultural sign systems of social cooperation—for sustaining health, for reproduction and for learning how to use environmental resources collaboratively (Sebeok, 1990; Trevarthen, 1990; Stern, 2010; Porges and Furman, 2011).

We relate autistic disturbance of cognitive functions to growth errors in creative agency attributable to events in brain development of embryo, fetus and infant (Trevarthen et al., 1998, 2006; Trevarthen, 2000; Trevarthen and Daniel, 2005; St. Clair et al., 2007). We address development of the autopoetic subcortical neurobiology that makes possible manifestations of intentions and emotions before birth (Delafield-Butt and Trevarthen, 2013), and the cooperation of movements after birth within an intimate infant-parent intentional system (Sander, 2008), which sustains itself by the primary emotional processes of consciousness (Solms and Panksepp, 2012). The motivation of the developing human organism is environment expectant, ready for sharing agency and emotions in movement, but this sharing is “anoetic”; that is, not dependent on acquired categorical knowledge of the structure and uses of the environment (Vandekerckhove and Panksepp, 2011). The infant is adapted physically and motivated psychologically to receive not only vital care in attachment to the mother, but also “companionship” for the young mind's growing purposes in imaginative movement and the uptake of new experience (Trevarthen, 2005, 2013). Shared health and meaning are created in human awareness by primary processes of joint agency and emotional sympathy between the movements of human bodies (Trevarthen, 1986b, 2012; Reddy, 2008; Stuart, 2010).

We need to have a clear conception of the nature of animal movement and its affective sociability if we wish to understand how children with autism fail to organize and time their movements effectively, hesitate to become affectively engaged with their parents as infants (Muratori and Maestro, 2007), and fall behind their peers in learning how to share and use knowledge of the human world playfully (Reddy et al., 2002).

Based on evidence of early neural growth errors in core brainstem systems during fetal ontogenesis, and on new evidence of disturbance of primary *prospective motor control* of expressive action, we present the following hypothesis on the etiology of autism for testing and argument:

1. A primary cause of autism spectrum disorders is an error in early growth of intrinsic motive and motor systems of the brainstem during prenatal ontogenesis.
2. This interferes with efficient integration of sensory information with motor timing, and is accompanied by disturbance of autonomic functions, disrupting timing and control of prospective sensory perception in movement as well as vital regulation of functions within the body. All these disorders become most obvious in early childhood, when a toddler normally gains many new powers of movement in engagement with the environment, including speech.
3. Social isolation, socio-emotional and cognitive delay, and language disorder in children and adults with autism are *secondary consequences* developed within socio-emotional systems as experience-dependent compensations for primary sensori-motor and affective integration errors and poorly regulated motor intentions. These compensations are elaborated mainly by cortical systems that grow after birth.

### Autism is a disorder of self-related motor-affective processes, which control development of shared cognitive representations

People diagnosed as autistic exhibit disabilities in regulation of the order and timing of moving, in the feelings of their bodies and emotional control, in selective expectation of objects for experience, in attention to other persons expressions, in the playfulness and humor of their social engagements, and in collaborative learning (Baron-Cohen et al., 2000; Reddy et al., 2002, 2010; Rogers and Williams, 2006; Mundy et al., 2009; Hobson and Hobson, 2011; Torres, 2013). cognitive disabilities attributed to failure in special modular mental functions of perceptual selection, of conceptual grouping, or of a capacity to conceive and think about the emotions behind other persons” face expressions, orientations and practical actions, or to imagine the representational contents of their minds (Baron-Cohen et al., 1985; Frith, 1989/2003; Morton, 2004), may only be identified after infancy. Similarly, definition of autistic disturbance by reference to neuropsychological tests that identify faults in praxis, gnosis, reasoning and language in adults after local brain injury ignores the large transformations in brain function and behavior that take place during psychological development (Karmiloff-Smith, 2009; Thomas and Karmiloff-Smith, 2002; Karmiloff-Smith, 2009).

We propose that faults in higher mind functions of persons with autism arise out of disorder in the early development of primary, non-reflective sensori-motor factors that regulate moving-with-awareness of an integrated Self. These affect vitality dynamics, the qualities of motor control that express essential expectancies of action and enable communication of emotion in purposes (Stern, 2010; Gowen, 2012; Gowen and Hamilton, 2013; Rochat et al., 2013). The primary processes of mental agency do not require conceptual representation or explicit reference to external events; they are primary conscious experience (Vandekerckhove and Panksepp, 2011). Growth errors found in formation of brain stem motor control and emotional systems of the embryo and fetus (Prechtl, 2001; Rodier and Arndt, 2005), interfere with the maturation of sensory-motor skills at significant periods in a child's early life, impairing cultural learning mediated in postnatal elaborations of the neocortex and dependent on creative emotional engagement with human company (Trevarthen et al., 2006). Interpreting autism in these terms requires attention to the environment–expectant processes of morphogenesis by which human bodies and brains are formed *in utero*, with special adaptations for intersubjective communication (Trevarthen, 2001a,b), and information on how additional brain networks grow and learn after birth (Thomas and Karmiloff-Smith, 2002). This is a “developmental psychobiology,” not a “developmental cognitive neuroscience” based on the neuropsychological definition of disorders inferred retrospectively from effects of damage to parts of the adult brain (Baron-Cohen et al., 2000). Psychological theory must also explain how individuals with high-functioning autism and Asperger's disorder perform certain feats of perception or action with remarkable precision, but with inadequate awareness of the context, or “weak central coherence,” in their self-related conceptions and plans for action (Frith, 1989/2003; Rinehart et al., 2001).

No single genetic, neurobiological or environmental factor has been identified as the cause of autism, which is also not attributable to the loss of a single cerebral function or capacity (Bauman and Kemper, 2005; Aitken, 2010). The complex and varied cognitive problems of people with autism, and the abnormalities in habits of action and of social response or use of language, are consequences of core disabilities, manifestations of which might be recognized, and compensated for, in infancy, before the development at the end of the first year of “joint attention” (Trevarthen, 2000).

A new scientific recognition of these core disabilities in autism, and their relationship to imagination for action and to qualities of movement, is emerging from attention to the emotions that evaluate other persons actions (Hobson, 1993, 2002/04; Reddy et al., 2002, 2010; Reddy, 2008; Hobson and Hobson, 2011), and from a brain science of intentions in movement and the intersubjective sharing of their dynamics of expression (Gallese, 2006; Stern, 2010; Gowen, 2012; Gallese and Rochat, 2013; Rochat et al., 2013).

### Autism compromises affective sharing, and requires creative response to this

When Leo Kanner (Kanner, 1943) distinguished “autistic disturbances of affective contact” in 1943, he accentuated that the disorder is emotional. Hobson and Hobson (2011) quote examples from Kanner's sensitive case studies that identify a difficulty in engagement with other person's intentions, experiences and feelings. Kanner also recorded that parents of these children were often concerned from the first year about their child's detachment or aloneness. Reddy (2008, 2011) cites a large number of studies that prove normally developing infants “know minds” and learn complex cooperative activities by deliberately engaging playfully and inquisitively with the way other persons display their interests, experiences and feelings. This eagerness for enjoyment of shared experience, a sympathetic activity, which goes beyond “joint attention to objects,” is weakened in autism.

The cognitive deficiencies of autism measured by tests of perceptual recognition, rational choice, and language are skills that must be gained by learned *accommodation* to objective experience, and normally depend on deliberate adult instruction. But all can be attributed to deep subjective causes that impair imaginative moving, the pleasures of the body in explorative action, and a motivation to deliberately share this “seeking” in inventive and playful, *assimilatory*, communication, going “beyond the information given” (Bruner, 1974). It appears likely that autism results from disorders of imaginative and sociable playfulness itself, for which the motives and emotions are apparent from birth. Such disorders can be traced back to creative developments of movement and awareness in body and mind before birth (Trevarthen and Delafield-Butt, 2013), to disorders of sensory-motor circular reactions that become the tools for mastery of engagement with the world (Piaget, 1951, 1954) and for the development of shared cultural understanding (Baldwin, 1902).

Though some medical treatments lead to improvements in associated conditions, there is no drug or surgical intervention for autism. A prescribed course of training or instruction in behaviors, cognitive abilities or communication by learned symbolic language may help, but can have adverse consequences, increasing the subject's anxiety, isolation and dependency (Trevarthen et al., 1998). Moreover, the activity, cognitive capacities, relationships and emotional well-being of a child or older person with autism can be improved by a variety of non-verbal, non-cognitive activities in which a therapist, who engages sensitively with the individuality of their impulses and felt experiences, accompanies the autistic person in the emotions of intimate engagement to more productive and less defensive states of activity and awareness. This type of relational and creative “art” therapy, which responds to and guides the primary actions, interests and feelings of individuals with autism, much as mother engages her affections with her animated infant from birth, can benefit language learning and both social and practical education (Malloch and Trevarthen, 2009; Stern, 2010).

Evidence that autistic behaviors express abnormalities of prenatal development of the brain stem (Rodier and Arndt, 2005) relate to evidence that early postnatal communication, if it is to support social and cognitive development, must be ready to protect the infant against autonomic reactions of protective withdrawal and depression, as well as to support positive initiatives promoting advances in social communication (Panksepp and Sahley, 1987; Panksepp and Watt, 2011; Porges, 2011; Porges and Furman, 2011). Infant psychology and paediatric practice have been transformed by abundant confirmation that precise coordination of well-formed intentions, interests and feeling may occur within the child and between the child and an attentive and affectionate adult from the neonate stage (Brazelton and Nugent, 1995; Trevarthen, 1977, 1998, 2009a; Stern, 2000; Sander, 2008; Nagy, 2011). This is the arena in which we must be alert for weaknesses in developing human sense and for special support it may need from the parental and social environment (Narvaez et al., 2013).

## Psychobiology of human mental functions

### Developmental neurobiology of self-conscious intentions with embodied feelings, and social awareness

Evidence concerning the generation of animate intentions, awareness and emotion in deep processes of the brain (Panksepp and Biven, 2012) questions the “thalamo-cortico-centric” theory of conscious awareness, thought and memory, which focuses on abilities that depend on learned definition of objects from information picked up *outside* the body, on the routines of fine articulate skills for using the environment, and on educated conventions of representation and reflective thought about objective information. Functional brain research shows that the primate neocortex is excited to regulate motor activities prospectively in reference to their goals, seeking perceptual confirmation by imaginatively simulating the completion of the action within an established context of multimodal information (Fogassi et al., 2005; Pezzulo et al., 2008; Pezzulo and Castelfranchi, 2009; Hesslow, 2012; Gallese and Rochat, 2013). The process of intending to act in a particular way is not a consequence of backward coupling of frontal cortex “executive functioning” to recollections of the past objects and events mediated impersonally in the temporal lobe. It is the product of a forward-looking creative imagination that builds an episodic memory of past events related to an intentional personal self (Tulving, 2002), with an autopoetic imagination equipped from the start with “implicit experiential and procedural memory processes that generate non-reflective qualia” (Vandekerckhove and Panksepp, 2011, p. 7).

These animating functions of the primate brain mediate intersubjective coordination of self-related experiences in intimate direct communication of purposes and feelings with others. The anticipations of experience are charged with emotional values linked in the brain stem with autonomic regulation of vitality within the body (Damasio, 2010; Solms and Panksepp, 2012), and these affections are communicated between subjects by a reciprocal *sympathetic* cooperation of purposes and experiences (not a one way imitation or shadowing of emotional processes now commonly called “empathy”). Human relationships and mutual awareness depend on relational emotions that promote social cooperation in performance of creative actions and thinking, to increase collective well-being (Stern, 1993; Hobson, 1993, 2002/04; Trevarthen, 2009a).

The well-coordinated performances and expressions of affect of newborn infants in expectant orientation to real or imagined objects, and to persons (Trevarthen, 1984, 1986b; Nagy, 2011), the development of intentional movements and rhythmic emotional expressions of fetuses (Trevarthen and Delafield-Butt, 2013), and the behaviors of anencephalic children (Merker, 2007) support phylogenetic evidence that primary conscious states and emotional evaluations, which are essential regulations in all goal directed consciousness, are indeed first generated and regulated sub-cortically (Solms and Panksepp, 2012), without neocortical involvement. These motor-emotional systems are elaborated in the orbito-frontal cortex and the temporal lobe of human beings, which continue to develop to adult stages (Schore, 1994, 2005). Before these developments they play a central role in maternal care, and in the repair of emotional disorders (Schore, 2003).

Affective self-regulation and emotional communication to regulate engagement with other individuals have evolved in vertebrates by elaboration of intrinsic neurochemical systems in the brain stem linked to the hypothalamus (Trevarthen et al., 2006). Regulation by the vagal nerve of essential self-related vital processes of heart activity, respiration and feeding is adapted for intersubjective coordination in the primate social brain by means of communication employing expressive movements of eyes, face, and vocalization. Throughout development of a child, from the time of maternal support of the infant through birth and nursing, there is a dynamic process that balances changes in self-regulation against the need for collaborative regulations of relationships with other persons in various degrees of intimacy (Porges and Furman, 2011; Carter and Porges, 2013). These have particular significance for identifying and explaining autism (Patriquin et al., 2013).

The importance of rhythmic emotionally expressive hand gestures in human communication from infancy (Trevarthen, 1986b; Trevarthen et al., 2011), indicates that forebrain systems for guiding action of the hands in complex manipulations have been recruited into the brain stem and limbic systems for assisting autonomic regulations by self-touching or holding and further adapted to the service of social coordination. Hands are part of the human emotional motor system (Holstege et al., 1996). Indeed, movements of “mimesis” for social celebration in dance and song, appears likely to have preceded evolution of speech and contributed to its power to communicate thoughts as *Homo sapiens sapiens* evolved (Donald, 2001; McNeill, 2005; Mithen, 2009; Gillespie-Lynch et al., 2013). The roots of this human talent for expressive gestural mimicry is apparent in infancy and an essential contributor to the intimacy of parental care (Trevarthen, 1999, 2013; Dissanayake, 2000).

Both gestural and linguistic languages develop in intense interpersonal communication mediated by vitality dynamics and expressions of emotional investment that provide a basis for the transmission of more differentiated semantic references by symbols (Stern, 2010; Lüdtke, 2012). Dynamic communications carried by consistent innate measures of moving in time (Pöppel and Wittmann, 1999), over intervals from fractions of a second to minutes and longer, are cultivated in all human societies in the arts of music, dance and theatre. They begin as a universal human regulation of rhythms of the mind or “biochronology,” active before birth and elaborated in the communicative musicality and rhythmic action games parents play with infants in the middle of the first year (Trevarthen, 1999, 2009b; Malloch and Trevarthen, 2009).

Autistic children show abnormalities in production and reception of communication by both speech and gesture, and in writing (Rapin and Allen, 1983).

### The neurology of communication by transfer of the dynamics and form of intentions and feelings in movement

New data from social neuroscience confirm the “common sense” that we are aware of other person's states of mind by immediate or direct engagement with the Other's *motor* intentions, by whatever modality or movement these intentions are expressed, matching them by instantaneous “affect attunement” (Stern, 1993, 2010) to the animation by which we generate intentions of our own Self (Gallese, 2006; Bråten, 2009). Sensitivity for the intentions, interests and feelings of other individuals, for the social affordances of their behaviors, must depend upon matching regulatory processes that govern the rhythm or pulse and expressive tonality or quality of movements of the human body as well as by “mirroring” their body-related form (Trevarthen, 1986b, 1999; Stern, 2010).

Regions in the adult cerebral hemispheres of a monkey or human being that are sensitive to organism-object relations, and that respond selectively to perceived *capacities for action* of the self, also respond to the possible actions available to, and enacted by others (Gallese, 2007). The same neural system is responsible for perceiving one's own possibilities for action *and* the possibilities for action of another. Direct *intra-personal* neural resonance within the “mirror neuron system,” reflecting the Self, gives one individual direct *inter-personal* access in “felt immediacy” (Bråten, 2009) with intentions in the mind of an Other made manifest in their body movement, in “intersubjectivity” (Trevarthen, 1979, 1998; Trevarthen and Aitken, 2001). Further, data from imaging of brain activities show there exists substantial overlap in activity of this system for awareness of actions with activity excited by merely *thinking about* an intentional act (Decety and Grezes, 2006).

Direct resonance between preparation, execution, observation and thought in action depends on “motor images” (Bernstein, 1967), which underpin perception, observation, and planning of goal-directed action, and also integrate Self-related experience (Llinàs, 2001; Northoff and Panksepp, 2008). An amodal perception-action system is also the means by which complex embodied human intentions may be communicated between agents across many channels of expression, in a “consensuality,” which, when further elaborated and mediated by language, becomes a tool for sharing abstract concepts and plans (Maturana et al., 1995).

Disruption of the neural systems of motor planning in time and space, by epigenetic dysregulation of early development in the brain stem, or by environmental insult to the growing brain, will have pervasive effects in maturation of consciousness, behavior and social engagement, such as occurs in autism (Aitken and Trevarthen, 1997; Trevarthen et al., 1998; Trevarthen, 2000).

### Prenatal genesis of autism

We have described the coordinative mechanisms in the brain as an “intrinsic motive formation” (IMF), “ready at birth to share emotion with caregivers for regulation of the child's cortical development, upon which cultural cognition and learning depend. … many psychological disorders of childhood can be traced to faults in early stages of brain development when core motive systems form.” (Trevarthen and Aitken, 1994, p. 597). The IMF, laid out in development of the fetus, is a core component of all of the sensory-motor mechanism of human communication—by gesture and dance, speech and song, or by writing, playing musical instruments and other manual or digital media (Trevarthen, 2001a,b). Rodier and Arndt (2005) relate autistic behaviors that limit expressive movements of the eyes, face and vocal productions, and anticipatory attention to expressive movements of other persons, to malformation in the embryo of core regulatory systems in the midbrain, the brain stem visceral efferent and afferent nuclei, and the olivary nuclei and cerebellum. They conclude, “there is no region but the brain stem for which so many lines of evidence indicate a role in autism” (Rodier and Arndt, 2005, p. 146).

### Imaginative intentions and emotions of the primary self

There has been, in the last two decades, a highly significant re-evaluation of the relationship between emotion and cognition, and their functional inseparability in human experience and in communication at all stages of development (Damasio, 2010; Panksepp and Biven, 2012). Comparative studies of the mammalian emotional system demonstrates that an *affective* core sense of the Self (Northoff and Panksepp, 2008; Solms and Panksepp, 2012) does not depend on learned conceptual knowing. This “anoetic” consciousness of a live body (Vandekerckhove and Panksepp, 2011) develops before a child becomes familiar with the external world through practice of intention and testing of actions which explore the affordances of situations and objects. At all stages of the development of human conscious intelligence this mobile self-with-feelings remains active, generating an innate spatio-temporal context for the arousal of movements to engage with the environment, and affective values for sustaining core vitality (Stern, 2010). From mid gestation through infancy the developing self is sensitive to other persons' responses to its activities and vitality, first showing signs of vital state to achieve shared “amphoteronomic” regulation of its own autonomics with those of the mother. After birth the infant signals its own rhythmically intended and affectively measured acts in responsive ways that lead to the “synrhythmic” communication for cooperative learning and cultural development (Maturana et al., 1995; Donald, 2001; Trevarthen et al., 2006; Malloch and Trevarthen, 2009; Porges and Furman, 2011).

## Development of human agency in infancy, and before birth

### Measures of infant sensory-motor intelligence, self-regulation and sociability

Movements of a baby under 2 months old are coordinated and integrated within a rhythmic awareness of a single intentional subjectivity (Trevarthen, 1979, 1984). These movements were described by Prechtl (2001) and Einspieler and Prechtl (2005) as “general movements” (GM), which, “involve the whole body in a variable sequence of arm, leg, neck, and trunk movements. They wax and wane in intensity, force and speed, and they have a gradual beginning and end. Rotations along the axis of the limbs and slight changes in the direction of movements make them fluent and elegant and create the impression of complexity and variability. If the nervous system is impaired, GMs loose their complex and variable character and become monotonous and poor.” (Einspieler and Prechtl, 2005, p. 61). General movements are not precisely focused, intentional and directed by discrimination of discrete objects, but they can orient head, eyes and limbs to external events in coordinated sequences within a body-related space (Trevarthen, 1984). Visually directed reaching in newborns compensates for changes in the “load” of a limb, which proves the responsiveness of this non-reflex imaginative coordination to proprioceptive reafference, or “body self awareness” (Van der Meer et al., 1996).

A newborn infant's movements are especially sensitive to sight, hearing and touch of an attentive the mother in face-to-face engagement, and they can take a creative part in a shared narrative of expressive action (Trevarthen and Delafield-Butt, 2013). Her voice was learned *in utero* (DeCasper and Fifer, 1980) and its sound motivates rapid visual learning of her face. Imitation tests, made with care to allow the infant to focus attention and regulate a state of responsive arousal, prove that a newborn can initiate eye-movements, face expressions, vocal sound patterns and hand gestures of another person (Meltzoff and Moore, 1977; Maratos, 1982; Field et al., 1983; Heimann et al., 1989; Kugiumutzakis, 1999; Nagy and Molnar, 2004; Nagy, 2011). These behaviors signaling a “second person other-awareness” are adapted for sharing curiosity for others' mental states of interest and affective appraisal (Reddy, 2011).

At 2 months, after a period of rapid maturation of sub-cortical and cortical visual-motor regulations of foveal sight (Trevarthen, 1986a), the infant's precisely timed responses of looking, smiling, and vocalization give evidence of preparation for sharing ritual practices and language (Bateson, 1979). Electroencephalic data on the activity of a 9-week-old infant's brain when looking at the photograph of a woman's face (Tzourio-Mazoyer et al., 2002) confirmed that complementary neocortical areas in left and right brain, which 2 years later will become involved in a child's learning of expression and reception of spoken language, are already components in cerebral regulation of interpersonal contact by a “social brain,” long before the training of a “social intelligence” by life with other persons (Frith and Frith, 1999). The subcortical visual and auditory systems that mature from the early fetal period show an asymmetry related to differences in left and right parts of the brain stem that mediate in complementary autonomic regulations (Trevarthen, 1996). Schore (1994, 2005) proposes that the early developing right brain motivates shared learning of perception and articulation of meaning in language when the left cerebral hemisphere shows an acceleration of growth in the second and third year, the period when diagnosis of autism becomes possible.

Developments around 3–5 months correlate with more differentiated movements of the baby's extremities when new neocortical sensory-motor functions are developing. Einspieler and Prechtl, label these subtle gestures “fidgety,” and describe them as, “small movements of moderate speed with variable acceleration of neck, trunk, and limbs in all directions” (Einspieler and Prechtl, 2005, p. 61). They lead the infant to make more discriminating orientations of head, eyes and hands intending to reach for and touch or take hold of objects at a distance from the body, and are accompanied by a fall in attention to the mother. This incites the mother to be more animated and playful, and to incorporate the baby's selective interest in objects into “person-person-object” games (Hubley and Trevarthen, 1979; Reddy, 2011).

### Programmed development of the infant-parent system

Longitudinal studies of developments in actions, perception and communication in the first two years, with information on internally regulated brain growth changes, confirm that there are transformations in the motives and emotions of the child for collaboration with parental care (Trevarthen and Aitken, 2003). Sander's studies of infants with their mothers from birth over the first 36 months showed that growth of a human life is sustained by a series of stages of adjustment within a system of human-to-human engagement (Sander, 2008). Both mother and child are significant actors, but in the creative process of development the child must normally set the pace and the times of important advance. Brazelton extended Sander's system approach to an interpersonal paediatrics accepting the conscious and personal powers of the newborn, and defining “touch points” in the developing life with parents and in the community (Brazelton and Nugent, 1995; Brazelton and Sparrow, 2006). Periods of change in developing powers that are both sensitive and significant, are symptoms of advances in motivation for learning and for communication (Johnson, 2005). Their consequences depend on collaboration with parents who are “attuned” to the infant (Stern, 2000), and both intimate and playful in their accommodation to the child's impulses.

Data from a review of the literature on changes in the child's psychology and brain over the first 18 months (Trevarthen and Aitken, 2003) point to natural emergence in the child of new levels of mastery of action and awareness at around 6 weeks, 4 months, 7 months, 9 months, and between 15 and 18 months. These agree with longitudinal studies of infant's capacity to take initiative in joint activities (Trevarthen, 1977; Hubley and Trevarthen, 1979; Reddy, 2011). These five advances in adaptive processes correlate with temperamental changes commonly referred to as “regressions.” They adapt to cultural differences in the frequency of parental initiatives or directives (Reddy et al., 2012). They are products of the active system of “intent participation” in the environment with companions that drive cultural learning (Trevarthen, 2013).

### Sensori-motor intentionality before birth: genesis of primary self-consciousness and the first intersubjectivity

Spontaneous movements develop in the late embryo and fetus, showing increased sensory awareness of their purposes (Delafield-Butt and Trevarthen, 2013). The first integrative actions of the nervous system are to move the body, and the first nerve tracts in the central nervous system are those that will activate movements to express different orientations and emotional states (Trevarthen, 1986a). After 8 weeks the core neurochemical systems of the subcortical brain that will link motor centers and select and evaluate experiences throughout life make their appearance. At this stage the fetus makes the general movements of Prechtl (2001). These become increasingly differentiated and controlled with the benefit of re-afference from sensory systems that grow in the following weeks. Detailed studies of by real-time ultrasonography demonstrate a fetus's exploratory sensation-testing to touch their own body, their face, the placenta, umbilicus, and the uterine wall with their hands at 11 weeks. They make jaw movements and swallow amniotic fluid, expressing pleasure or disapproval at tastes, sucking and smiling or grimacing with disgust. Complex movements of trunk, arms, and legs position the body, and may react to movements of the mother's body and to the contractions of the muscles of her uterus (Lecanuet et al., 1995; Trevarthen et al., 2006; Piontelli, 2010). In weeks 10–14 fetal movements become differentiated into individual, isolate actions with increasing goal-direction to particular parts of the body (Prechtl, 2001; Piontelli, 2010). The arms and hands “test” sensitive zones of the body, especially to the face and head, exploring the border of sensory innervation on the top of the head (Piontelli, 2010, p. 61–67).

In singleton pregnancies motor planning of action patterns adapted for different goals is evident before 22 weeks gestational age (Zoia et al., 2007). In twin pregnancies, movements directed by one twin to the other are “carefully” slowed, even by 18 weeks, which the researchers interpret as evidence of a primary “social awareness” (Castiello et al., 2010). At this time the motor centers of the brain stem and spinal cord are directing the coordinated behavior of the fetus (Okado, 1980). Neocotical cells do not develop dendrites until after 26 weeks of gestation (Hevner, 2000).

This natural history of human movement at a stage of development when the sensori-motor environment can only be the properties of an organized body itself appears to support Lashley's conclusion that propositional thought may depend on, and indeed be derived from, the spontaneous syntactic ordering of movement sequences (Lashley, 1951, p. 122). The fetus has an imaginative “motor intelligence” and can formulate orderly projects without neocortical skills.

Expressions in fetuses, in addition to twisting movements of distress and tentative exploration by touch, give evidence of emotions—of discomfort, curiosity or pleasure, adapted for communication of interests and feelings. In the third trimester, movements of the face visualized by 4D ultrasound develop into complexes that define a “cry face gestalt” or a “laughter gestalt,” expressing emotions that will communicate powerfully immediately after birth in the regulation of parental care (Reissland et al., 2011). Maternal hunger with depletion of energy supply to the fetus drives “anxious” patterns of fetal movement. The mother and the fetus are already affectively connected. These discoveries prompt a revolution in psychological theory and medical ethics. There is a consensus in modern paediatrics that by 24 weeks the fetus should be considered a conscious agent deserving the same standard of sympathetic medical care as adults (Royal College of Obstetricians and Gynaecologists, 2010).

### Readiness for support of the body in rhythms of movement, aware of surroundings, and attentive to human company in movement

Infants demonstrate the regulations of an innate time for life in movement. Research on their dynamics and coordination with a parent's movements have led to a natural science of human “musicality” (Trehub, 1990; Papoušek, 1996; Malloch, 1999; Malloch and Trevarthen, 2009). Inspired by discoveries of precise analysis of films, revealing self-synchrony of movements of individual actors and inter-synchrony between actors in conversations (Birdwhistell, 1970; Jaffe and Felstein, 1970; Condon and Ogston, 1971) researchers found that infants and adults share matching rhythms (Condon and Sander, 1974; Beebe et al., 1985; Jaffe et al., 2001). One remarkable video recording made by Saskia van Rees of a 2 month premature infant in precisely timed coordination of dialogue of simple “coo” sounds vividly demonstrates how this shared sense of time for combining syllables in phrases may lead to a narrative in wordless dialogue (Trevarthen, 1999).

Two bands of time are shown to be fundamental in dialogues, games and songs between young infants and their parents (Trevarthen, 1999, 2009b). Faster rhythms of syllables and phrases in speech and song, or dancing steps and gestures, correspond with arm and hand grasping for object manipulation, or of the head and eye rotations that perform visual inspection. These range from the median syllable frequency of 1.5–3 per second—the same as a running or fast stepping, a glance or eyebrow rise, a laugh or a hand wave—to every 3–5 s for a visual scan, a manipulative sequence, a phrase of speaking or song, and a cycle of deep breathing. These are somato-motor coordinations that achieve use of the environment and pickup of information for perception, or of a communicative message, in the “psychological present,” the “here and now” of consciousness in action.

Slower periods of sensed vitality, as expressed in the “extended present” of an episode in a story, a verse of singing or a stanza of poetry, occupy 10–25 s. Longer times of imagined activity and narrations form natural elements of 25–50 s in the rhythmic verses, playful or calming, of baby songs in all languages. These slower events are identified with autonomic events that regulate arousal, hunger and wakefulness throughout life, and regulation of the rate of heartbeat and breathing by the vagal nerve (Delamont et al., 1999). They are accompanied by bursts of electrical activity in the cerebral cortex that have a role in the fluctuating experiences of dreaming. They link the imagination with the economy of life energy in the body, and with the expressive arts.

Stern (1993, 2000, 2010) called the cycles of arousal or variations in vitality dynamics in mother infant play “emotional narratives” expressing “implicit relational knowing.” Malloch analysed the controlled patterns of change in voice qualities and pitch of the voices of mothers and infants in dialogues and baby songs as “narratives” that, “allow two persons to share a sense of passing time, and to create and share the emotional envelopes that evolve through this shared time. They express innate motives for sharing emotion and experience with other persons and for creating meaning in joint activity.” (Malloch, 1999, p. 45). These shared “routines” are identified by Bruner (1999) as the medium for reference in language. We have recently been finding evidence of the same “narrative” cycles of arousal in the “general movements” of newborn infants, which may be shared with a sensitive mother who coordinates with her baby by modulated vocal sounds, touches or rocking. They participate in tides of consciousness of being together that later will regulate the changes of meaning in a story or the recollections of episodic memory (Delafield-Butt and Trevarthen, 2013; Trevarthen and Delafield-Butt, 2013).

## Sensori-motor dis-coordination in autism, from infancy

### Deficit in prospective motor control in autism and its consequences for developing intentionality and learning

The complex disorder of childhood autism, and how it has serious effects on a young child's life, may be described as follows:

“By about one to two years after birth … at a time when infants usually become acutely aware of other people and what they are doing, full of playful imagination and eager for new experiences, these babies became strangely self-contained or isolated in their own world and increasingly unresponsive or irritable, and difficult to understand; their vocalizations movements often seemed repetitious and pointless, and their gestures and postures were also odd. Throughout their childhood they continued to express themselves in ways that made parents, teachers and other children feel unable to make contact.

As pre-schoolers, the children are not insensitive to others or unaffectionate, and they can show strong likes and dislikes for particular people. Sometimes they imitate or seek to interact, but never in a free and easy way, and sometimes with a peculiar ritualistic insistence, and remarkable inattention to their effects on other people. Strange postures and movements and a need for sameness, combined with obsessive interest in certain objects and experiences, cut them off from others. At times they seem to be in a trance, “floating off,” “looking” or “listening” when nothing is there, often with strange flapping of the hands, or an enigmatic smile, and they only make unintelligible baby-like vocalizations. They may get into inexplicable panics and seem very distressed, anxious or terrified, especially when forced to have close contact with people or in strange environments. In general they do not like, or fear, unfamiliar places or routines. They protest at irregularities in their world and repeat seemingly trivial actions for their own interest. Some, in panicky states or anger, may injure themselves. Most of the time, however, they seem content to amuse themselves, often performing favorite actions over and over. Their behaviors can be frightening and distressing to parents who need help to understand what is wrong and how to cope with a child who looks healthy enough, but who won't respond.”

(Trevarthen et al., 1998, p. 1–2).

Odd behaviors like these are seen in children who do not have autism, but they are momentary and easily regulated by the child's playful resourcefulness or by affectionate attentions of parents, and in shared enjoyment with other children. The autistic child has persistent problems in both self-regulated actions and emotions, and in awareness of other person's intentions, interests and feelings. There are conflicting ideas on the causes of these problems and how to respond, especially for the early stages.

Disorders of movement in children with autism particularly affect expressive movements in communication (Ricks and Wing, 1975; Damasio and Maurer, 1978; Gillberg and Coleman, 1992; Frith and Frith, 1999; Oller et al., 2010). These have lead to an interpretation in terms of a deficit in “executive functioning” (Rumsey, 1985) attributed to a developmental fault in the frontal lobes that manifests itself in the second year. Recent data point to a more basic and probably earlier developing deficit in prospective control of movements (Mari et al., 2003; Rinehart et al., 2006a; Dowd et al., 2012; Gowen and Hamilton, 2013). For example, in an automated vocal analysis of a large body of data recorded from natural expressive behavior of infants 10–50 months of age, Oller et al. (2010) identified massive delay in development of movements of vocal articulation in children developing autism or language delay. Such disorders affecting communication behavior can be explained as originating as faults in the timing and integration capacities of the brainstem sensorimotor system, which develops prenatally and affords prospective control for later developments in psychological functions. Failure in cognitive strategies of “action planning” and “action execution” (e.g., Rinehart et al., 2001; Nazarali et al., 2009) attributable to change in mirror neuron systems (e.g., Cattaneo et al., 2007; Fabbri-Destro et al., 2009), require higher-order cortical processing, which develops after birth.

Children with ASD differ from typically developing children in the efficiency of three types of prospective motor control:

- Generation of *single actions*, such as when extending the hand to touch, or indicate, an object of interest;
- Organization of a *series of actions* to perform more complex tasks or projects, including speaking, and
- Simultaneous *coordination of multiple action units* to achieve coherent purpose, as in postural accommodations when standing or walking.

Simple “action units” and serially organized “action chains” both require precise coordination of muscle actions that are conceived or imagined “ahead-in-time” so that they achieve a desired future effect efficiently (Bernstein, 1967; von Hofsten, 1993; Lee, 2009). And an integrative control of movement is a necessary foundation for learning more advanced and complex tasks, such as speaking and reading (von Hofsten, 2004, 2007). Awareness of others' intentions requires detecting prospective control in their movements, and this is apparent in how infants participate in dialog and games (Trevarthen, 1986b). Failure to time movements prospectively and meet expectation in movement will thwart efficient goal acquisition, confuse awareness and frustrate a sense of success, causing negative emotions of self-protection and avoidance (Bower et al., 1970; Rovee-Collier et al., 1978).

- *Evidence for disturbance in prospective control of single action units*.
  Autistic persons exhibit significant differences in the timing and patterning of single movements (Rinehart et al., 2001, 2006a; Mari et al., 2003; Nazarali et al., 2009; Dowd et al., 2012). The type of disturbance varies with the task and the sub-group examined. For example, in a reach-to-grasp task individuals with ASD grouped by low or average to high intellectual ability, with full-scale I.Q. scores below and above 80, exhibited different kinematics, and both groups acted significantly less efficiently than typically developing children (Mari et al., 2003). Differences between ASD groups were thought to reflect different compensatory coping strategies for a primary deficit in motor planning. The autistic individuals also failed to coordinate the two sub-actions in the reach-to-grasp task, i.e., reaching of the arm and the opening of the fingers. They performed one act and then the other separately. Typical children coordinate the sequence of arm and hand actions in “pre-reaching” and gesturing fluently from early infancy (Trevarthen, 1984; Rönnqvist and von Hofsten, 1994; Prechtl, 2001).
- *Evidence for disturbance in serial organization of multiple action units*.
  The progressive planning of “action chains” communicate intentions. When we see someone grasping a bottle, for example, the initial reaching movement of the arm differs depending on whether the goal is to shelve it or to serve some wine (Jeannerod, 1999). The postural preparation of the body and extension of the arm, with shifts of gaze, are adjusted from the start in different ways depending on the final goal. Children with ASD have deficits in this preparatory coordination for motor sequencing or action chaining (Cattaneo et al., 2007; Fabbri-Destro et al., 2009). Typically developing children, when asked to perform an object manipulation task, such as turning an upside-down drinking glass right-side up, adjust their body posture at the start of the action so that their final posture is comfortable (Rosenbaum et al., 1990). Children with autism begin with a comfortable posture and conclude it in an uncomfortable one, suggesting a deficit of motor “knowledge” of how the action will proceed.
  Cattaneo and colleagues (2007) used electromyographic recordings of the mylohyoid muscle movements that lower the jaw and raise the tongue for reaching-to-grasp-to-eat, and they compared this sequence with the muscle activity during a movement of reaching-to-grasp-to-place. They found that typically-developing children anticipated eating the food with mylohyoid activation beginning well before their hand had grasped the piece of food. In contrast, this activation did not start in children with ASD until the food was already grasped in the hand and traveling toward their mouth, demonstrating a failure to couple the action chains efficiently. This lack of anticipation was also evident when the children were asked to watch another person perform the reach-to-grasp-to-eat action. The mylohyoid activation occurred in typically-developing children at the onset of the other's movement toward the food, but in autistic individuals there was no mylohyoid activation at all.
- *Evidence for failure in simultaneous integration of multiple action units*.
  Measurements of children's postural adjustments and muscle tensions during load shifting shows that prospective control of whole-body posture and perception of body-space goals, which require synchronizing and co-ordinating action units throughout the body in shifts of the legs, chest, back, and arms, are also disrupted in autism (Schmitz et al., 2003). Disturbances of prospective control for the whole body are confirmed by data on gait differences in individuals with autism, showing an increase stride length and variability of the width of stride, but also significant differences in postural adjustments of the upper-body to maintain balance (Hallett et al., 1993; Vernazza-Martin et al., 2005; Rinehart et al., 2006b; Calhoun et al., 2011; Nayate et al., 2011). They also have difficulties in perceiving the environmental context for their movements (Gowen and Hamilton, 2013).

### Differences in prospective motor timing affect social expectation and understanding

The subtle deficits in prospective motor control of children with ASD must be involved in the symptoms of social isolation and emotional distress that they show. They have difficulties in communicating their intention in gestural acts, and in sensing the dynamics of another's intentions from their movements (Cattaneo et al., 2007; Zalla et al., 2010; Gowen, 2012). Imitation-based or interaction therapies for ASD employing sensitive response to signs of intended movement are able to assist because they facilitate both anticipation of actions and psychological and emotional connection (Escalona et al., 2002; Nadel, 2006; Zeedyk, 2008; Field et al., 2011; Solomon et al., 2012). The therapist acts to excite anticipation, which simplifies and supports the performance of desired actions. It also explains why insistence on evidence from repeated measures of performance in tasks to test perceptual preferences or cognitive mastery can fail to detect or explain the cause of failure (Wigram and Gold, 2012). Such external measures, focusing on achievement of goals or response to facts, neglect the temporo-spatial phenomena of prospective motor control within the subject.

Problems of intentionality and its perceptual guidance in autism, and pathological defense against sensory overload (Rosenhall et al., 1999; Foxton et al., 2003), may be due to faults in motor regulations of sense organs; of the inner ear to adjust the sensitivity of hearing, and of head and eye movements to control selection of detail by foveal fixation which is guided by pick-up of global information from the ambient field. Hearing and production of speech sounds, which autism impairs in differing degrees, is particularly demanding, requiring detection and control of affective expression transmitted by small modulations in the timbre, pitch and loudness of vowel sounds, and their constraint by consonants produced in rapid sequences to articulate intelligible words in information-rich phrases. Autism, however, interferes not only with the motor controls of selective hearing and seeing, but with attention to all the expressive movements of other persons.

In high functioning persons with autism, exceptional abilities in detecting, separating and combining visual details or pitches of sounds (O'Riordan et al., 2001; Bonnel et al., 2003; Mottron et al., 2006) may be a consequence of compensatory hypertrophy in higher cortical sensory systems driven by a bias to detect affective self-related feedback or support. Ockleford's experience with supporting exceptional performative talents in autistic children who cannot speak suggest that pleasure from control of pitch in sounds from musical instruments activates a primary reward system different from that which discriminates speech components (Ockleford, 2012, 2013). In confrontation with another, a person with autism avoids looking at the eyes, directing attention to the mouth (Senju and Johnson, 2009). Given that rapid movements of the eyes transmit important information about the direction and intensity of *interest*, in preparation for shifts in locomotion, posture or reaching by hand, as well as selective attention to individuals in a group, they implicate tracking of sequences of intended action to engage with others' prospective control in thought and action (Bal et al., 2010). Lower face expressions and mouth movements express *affect* and are essential for emotional sympathy. They attract attention of an observer for judging another person's feelings.

Failure to appreciate playful teasing and humor and avoidant or defensive reaction to strangers, as well as preference for familiar surroundings and consistency in placement of objects or execution of routines, characteristics of ASD, all point to a disturbance of imaginative curiosity for prospects of action. They are as much disorders of self-regulation of pleasurable movement-with-awareness as of affective other-awareness, and they impair intentional and emotional engagement (Hobson and Hobson, 2011; Reddy, 2011)

### Disorders of autism in the first year

Teitelbaum and colleagues (1998, 2002), studying home movies of infants later diagnosed as autistic, made a comparative analysis of the developmental stages of turning over, crawling, sitting, standing and walking, which infants typically master in the first year. Using the Eshkol-Wachman Movement Notation for temporal and spatial parameters of human body movement they showed deficits in whole body control and sequencing of the movements of trunk, head and limbs to control balance and posture changes, which were interpreted as disordered sensory-motor reflexes. These detailed observations have been helpful for parents who suspect their infant may be developing autism, assisting them to engage the attention of medical specialists and therapists (Teitelbaum and Teitelbaum, 2008)

Similar disturbance of anticipatory regulations of whole body postures were found by Danon-Boileau (2007) in films made of two sisters while they were being bathed by their mother; one, at five months, who later developed autism, and the other who developed normally, at 3 months. The films show the anxiety and awkwardness of the first girl who scarcely looked at her mother, and an analysis of the mother's speech shows she was not “in contact” and was using her voice with a detached tone, to draw response. With the normally developing sister the mother's speech is lively and addressed to the child as person seeking to share the experience. This infant keeps eye contact with the mother and reacts expressively. Similar observations were made in an analysis of home movies of identical twin girls at 10 months, when their father was helping them to walk or playing a game with them in the family living room (Trevarthen and Daniel, 2005; St. Clair et al., 2007). One girl later diagnosed as autistic, and who did not speak until the age of 3, showed clear delay in motor coordination for stepping and for regulation of her sitting posture. She lacked attention to other persons' eyes and made fleeting smiles and she could not participate in a teasing game with her father that required anticipation of his rhythmically phrased behaviors and speech. The rhythms and expressions in response to teasing and tickling with the father were different from those of the typically developing twin, and the father was unable to reciprocate, creating confusion in games and interactions. Her sister who had a mild retardation at school age, developed normally through the first years showing no evidence of autism.

The lack of responsive attention by the infant developing autism to her father's attempts to play caused him to become irregular and insistent in his solicitations, which afterwards he could see only confused the child. The same transformation of parents' responses to avoidant or disengaged behavior of an infant developing autism have been noted in other studies of home movies and in prospective studies of siblings of autistic children, i.e., a change to a more insistent and monotonous mode that tries to excite a response (Baranek, 1999; Saint-Georges et al., 2010, 2011). For example, there is a lack of the affective modulation of the parent's voice in speech to an infant who later develops autism (Mahdhaoui et al., 2011). Disorder in development of the child's vocal control on the way to mastery of speech, such as that demonstrated by Oller et al. (2010) for the crucial period from 1 to 4 years, will affect the parents ability to share talking, and prompt them to use stimulating or coercive ways of engaging with the child.

Two research strategies have been used to search for evidence of abnormal development before medical diagnosis is possible: prospective study of the infant siblings of older children with autism. The two procedures confirm important conclusions about manifestations of autistic disorder that are developing in the first 18 months after birth (Zwaigenbaum et al., 2005; Saint-Georges et al., 2010). They highlight effects of the “flatness” and lack of seeking for engagement and also changes associated with the phases of motor development which were recorded by Teitelbaum (Teitelbaum et al., 1998, 2002), and the development of interest in objects. Attention to objects was normal in the first six months in infants developing autism when their attention to social engagement was significantly low (Maestro et al., 2002). There is a specific loss of interest in other persons” expressions early in infancy (Muratori and Maestro, 2007).

Expression of intentions and affects is achieved with cross-modal fluency between voice and gesture that promotes sympathetic action and shared experience with “affect attunement” (Trevarthen, 1986b, 2009a; Tronick, 1989; Stern, 2000; Reddy, 2008). Expressive acts, like all goal-directed voluntary movement, require prospective control, and by assimilation of the form and flow of the movements of the body and voice of one subject states of intention, affect, arousal and interest are conveyed to the awareness of the other in “felt immediacy” (Bråten, 2009; Stern, 2010; Trevarthen et al., 2011). If predictive control of the timing and harmonization of these expressive body movements are disrupted, then psycho-motor attunement with the perceptual and motor experiences of others will be confused.

Magnetic resonance imaging of the brains of autistic children indicate reduction in size of the brainstem and midbrain at birth, a loss of tissue more than compensated for by excessive growth of the brain as a whole postnatally (Hashimoto et al., 1995). Detailed neuroanatomical investigation of brains from children with ASD also indicate limbic midbrain structures and brainstem regions are affected (Rodier and Arndt, 2005). Of particular note is an abnormality in the inferior olivary nucleus, a prominent lower brainstem nucleus known to be involved in perceiving and controlling of the timing of movement (Welsh et al., 1995), indicating a likely primary site of disruption underpinning ASD motor deficit (Welsh et al., 2005).

The data on motor impairments in ASD and their early manifestation in infancy confirm a primary deficit in the capacity to perceive and move the body in a planned way, which limits the capacity to control the timing of actions of the body and their perceptual consequences, and thence impairs the communication of intentions and ideas.

## An interactive relational approach to therapy and teaching, nurturing intimacy and creativey of movement

“Musical structure in improvisation can provide a framework for creative development, and … more creative skills may well-emerge given a structure than one might see from a purely free form of improvisation—where a lack of direction and model may leave the “non-musician” client struggling to find out how they can “create” music…. Creativity is a key process in improvisational music therapy, and demands substantial skill and flexibility in the therapists to nurture in clients for therapeutic benefit.”

(Wigram, 2006).

Interactive music therapy for both diagnosis and treatment of autism indicates that the aim of a therapist or teacher is to provide support for creativity, and that this requires both a “direction and model” and “skill and flexibility.” It requires a guide that protects the learner from “struggling to find out how they can create.” And it requires descriptive evidence from single case studies (Wigram and Gold, 2012). In the controversial field of therapy for children with autism there is a bewildering range of theories and advice for procedures, which range from strict teaching of skills to control disordered actions and feelings and to coax communication, to permissive environments where possible distractions are eliminated and attempts are made to give comfort (Trevarthen et al., 1998; Teitelbaum and Teitelbaum, 2008). Given the evidence that the core deficit in autism concerns prospective sensori-motor control and affective self-regulation, especially for activities of communication, we focus our final comments on evidence that intimate or intensive engagement with the impulses of affected children in ways that bring pleasure from control of actions and mutual recognition may bring benefit for creative learning of practical skills and artificial rituals of shared experience, including language.

Finely measured pulse, form and flow of the enactments of the sensuous body and voice convey psychological states of intention, affect, arousal, and interest (Trevarthen, 1986a,b; Stern, 2010; Trevarthen et al., 2011; Hardy and Blythe LaGasse, 2013). Gestures made in communication are controlled and directed in body-space and by selecting transitory goals with precise timing of muscular energies that display affective content in “narrative” sequences (Schögler et al., 2008; Trevarthen and Delafield-Butt, 2013). It follows that, if the common control of body movements is disrupted, then the individual will have difficulty finding psycho-motor attunement with the perceptual and motor experiences of typical others.

Understanding of the fundamental and deeply felt disorder in autism as failure of integrative brain activity for carrying out sensori-motor intentions with ease and creativity, that it is a disorder that also affects communicative expression and perceiving the motor intentions of others, may help explain how intensive, imitation-based therapies attentive to emotions may be effective and may foster enjoyable response and interest (Nind, 1999; Field et al., 2002, 2011; Nadel, 2006; Nordoff and Robbins, 2007; Zeedyk, 2008; Caldwell, 2010; Frank and Trevarthen, 2012; Lüdtke, 2012; Solomon et al., 2012). By consciously “attuning” to the motor acts of the autistic patient and feeling their affective and intentional content in “intense interaction,” before re-enacting creative collaborations with adaptation to responses, the therapist provides an exterior pattern of actions that are timed and directed sensitively to compensate for repetition of uncertain, anxious attempts (Hardy and Blythe LaGasse, 2013). A responsive, “listening” makes communication possible, as well as progress to new self-confident and joyful experience, which may free an exceptional talent (Ockleford, 2013).

Sensorimotor attunement in therapy embodies mental/affective components as much as it does the motor expression, and in so doing is able to open up a co-regulation of arousals, interests, and intentions in a person otherwise unavailable and isolated. All movements are considered valid expressions of purposeful states, and even stereotypies are regarded as affective sensori-motor acts capable of initiating communication, not disregarded an unintentional, non-mental motor acts. As the therapist attends to the movements of the person, attuning to them with her own body movements, so they begin to generate an implicit, affective, and inter-subjective psycho-motor connection. Such therapy can aid not only the autistic child to achieve communication, but can be of great help to a parent. It may bring an autistic person of any age and to more self-confident and articulate participation in an intimate community of knowledge (Frank and Trevarthen, 2012; Lüdtke, 2012).

It is the experience of any therapist who works with persons suffering from autism that a conscious care must be taken to “stand back” and allow any impulse the child or adult may show to take its course, indeed shadowing or mirroring it to aid its motivation. This is the principle put into the practice of interactive music therapy (Robarts, 1998; Wigram and Gold, 2006; Nordoff and Robbins, 2007; Wigram and Elefant, 2009; Ockleford, 2013). A more explicit standing back, called “asocial,” is practiced by the method developed by the paediatric neurologist Waldon to assist persons with a wide range of disabilities in acting and thinking. The therapist places him or herself behind the client, holding the arms to guide the hands in performance of tasks to move objects in such a way that a goal or project is completed bringing a sense of satisfaction. This method has proved effective in helping young children overcome the confusion and isolation of autism in a way that makes productive and progressive motor learning possible (Solomon et al., 2012).

### Conflict of interest statement

The authors declare that the research was conducted in the absence of any commercial or financial relationships that could be construed as a potential conflict of interest.
